# Evaluation of soft and hard tissue responses to dental implants placed with and without platelet-rich fibrin

**DOI:** 10.6026/973206300215006

**Published:** 2025-12-15

**Authors:** Mohammed Ahsan Razi, Parveen Ranga, Rakhi Kumari, Amit Kumar, Ria Sahay, Sharmila Kumari

**Affiliations:** 1 Department of Periodontology, Hazaribag College of Dental Sciences & Hospital, Hazaribag, Jharkhand, India; 2 Department of Dentistry, Adesh Medical College and Hospital Shahabad Markanda, Kurukshetra, Haryana, India; 3 Department of Dentistry, Desh Bhagat University, Mandi Gobindgarh, Punjab, India; 4 Department of Periodontology, Dental institute, RIMS, Ranchi, Jharkhand, India

**Keywords:** Bone density, gingival health, implant survival, Platelet-rich fibrin, soft tissue healing

## Abstract

The challenge of enhancing soft and hard tissue healing during dental implant procedures, particularly in terms of preventing implant failure and improving tissue regeneration, which is explored through the use of Platelet-Rich Fibrin (PRF). The study population consisted of 60 randomly selected participants receiving either treatment with PRF or without PRF. The clinical and radiographic data were compiled at Baseline, 1, 3 and 6 months post-operative period respectively. Results show PRF positively impacts on the health of the gingiva preserve bone levels and improve survival rates of the implant. Thus, we show the understanding of Platelet-Rich Fibrin (PRF) as an adjunct in dental implant procedures, highlighting its positive impact on both soft and hard tissue healing, which may improve clinical outcomes, including gingival health, bone preservation and implant success rates.

## Background:

The placement of dental implants is an effective restorative method to replace missing teeth. Over the years, dental implants have transformed restorative dentistry [1]. They have provided patients with an improved level of dental function, facial aesthetics and an enhanced quality of life [2]. However, the success of dental implants depends greatly on the process of osseointegration, the biological behavior of the surrounding tissues and the bone quantity and quality at the site of implant placement [3]. Though dental implants overwhelmingly succeed, complications of closing the tissues implant failure and peri-implantitis are a reality of clinical practice. This is why the refinement of techniques and materials is endlessly pursued to improve the outcome on the patients [4]. An example of an innovation is Platelet-Rich Fibrin (PRF), a second-generation platelet concentrate. Derived from a patient's blood, PRF is later used for wound healing and tissue regeneration due to its healing factors and cytokines [5]. Fibrin, plates and leukocytes factors constitute PRF and it is used for soft and hard tissue healing. In dental implantology, PRF is used during implant placement due to its potential to enhance faster and more effective osseointegration, tissue regeneration at the implant site, reduce infections and promote tissue regeneration around the implant [6]. Recent research suggests that the use of PRF in dental implant placement enhances the healing tissue response and soft tissue over implants placed without PRF [7]. This is because PRF platelets are activated, which release growth factors that stimulate the healing of both soft and hard tissues in the body through cell multiplication, angiogenesis and collagen synthesis [8]. Also, PRF used is more compatible in the body and safe to use compared to synthetic grafts. Synthetic grafts are associated with complications and side effects [9].

The importance of osseointegration during the healing of hard tissue cannot be overstated, as it remains crucial for the long-term success of dental implants. Only with successful osseointegration will an implant provide the necessary stability and durability for the bone-enclosed prosthesis [9]. In the case of soft tissue healing, its successful completion is necessary in order to stave off infections and peri-implantitis, conditions which may result in the loss of the implant. Under the right conditions, the use of PRF will advance the healing of both hard and soft tissues, resulting in improved clinical results [11]. That said, even with the suggested benefits of PRF, understanding its implications and the extent to which it can improve the outcomes of an implant will continue to require extensive research [12]. Many studies demonstrate positive results; however, no consensus exists regarding the optimal use of PRF in dental implantology, particularly regarding its timing, overall impact on implant longevity and patient outcomes [13]. Therefore, it is of interest to determine the effects of dental implants placed with versus without PRF on both soft and hard tissue responses.

## Methodology:

The present study was conducted at Department of Periodontology & Oral Implantology, Hazaribag College of dental Sciences & Hospital, Hazaribag, Jharkhand, India. This study aimed to evaluate the soft and hard tissue responses to dental implants placed with and without the use of Platelet-Rich Fibrin (PRF). A prospective, randomized controlled trial design was employed, with a total of 60 participants who were randomly assigned to two groups. The first group (30 participants) received dental implants placed with PRF, while the second group (30 participants) received implants without PRF. The outcome measures assessed both soft and hard tissue healing, including clinical and radiographic evaluations. The study included 60 participants, with the inclusion criteria being: adults aged 18-65 years, individuals requiring single or multiple dental implants, adequate bone volume as determined by radiographic examinations (e.g., CBCT) and good general health without contraindications to surgery. Exclusion criteria included pregnant or breastfeeding women, individuals with severe periodontal disease, smokers (those smoking more than 10 cigarettes per day) and patients with systemic conditions such as uncontrolled diabetes or immunosuppressive treatments. The participants were randomly divided into two groups using a computer-generated randomization list. Group 1 received dental implants placed with PRF and Group 2 received dental implants without PRF. For both groups, the implant placement procedure followed a standardized protocol. The surgeries were performed by experienced Periodontist & Oral Implantologists under sterile conditions and local anesthesia to ensure patient comfort. Initially, clinical and radiographic evaluations, including CBCT scans, were conducted to assess bone quality and quantity. A blood sample (approximately 10 ml) was drawn from each participant for PRF preparation in Group 1.

For Group 1, the blood sample was made according to the Choukroun's method, collected about 10 ml of whole venous blood into sterile vacutainer tubes without an anticoagulant. We Centrifuged these tubes at 3000 rpm (800g) for 10 minutes. Prepared PRF was applied to the implant site after placing the implant fixture, covering the surrounding soft and hard tissues. In contrast, Group 2 underwent the same implant placement procedure but without the addition of PRF. The implants used in both groups were titanium-based, known for their optimal osseointegration properties. Following implant placement, all participants received the same post-operative care, including antibiotics, analgesics and oral hygiene instructions to minimize the risk of infection. Regular follow-up appointments were scheduled at 1 week, 1 month, 3 months and 6 months post-surgery to evaluate wound healing and assess any potential complications. The primary outcome measures included assessments of both soft tissue and hard tissue healing. Soft tissue healing was evaluated using the Gingival Index (GI) and Plaque Index (PI), which assessed the health of the tissues surrounding the implant, including inflammation, redness, swelling and bleeding upon probing. Attachment Level (AL) was also recorded at baseline, 1 month, 3 months and 6 months post-surgery to monitor soft tissue stability. Additionally, peri-implant soft tissue thickness was measured using a periodontal probe. Hard tissue healing was assessed using radiographic analysis. Standardized periapical radiographs were taken at baseline, 1, 3 and 6 months post- surgery to evaluate bone density and the surrounding bone of the implant. A CBCT scan was also performed pre-operatively and at 6 months for a more detailed analysis of bone remodeling and osseointegration. Bone level measurements were made by determining the distance from the implant platform to the first bone-to-implant contact on radiographs. Bone density was evaluated using Hounsfield units on the CBCT scan to assess any changes in the surrounding bone over time. The data collected were analyzed using appropriate statistical methods. Descriptive statistics (mean, standard deviation, frequency distributions) were calculated for all variables. Chi-square tests were used for categorical variables, while independent t-tests or Mann-Whitney U tests were used to compare continuous variables between the groups. A p-value of less than 0.05 was considered statistically significant.

## Results:

The data were analyzed across multiple time points (Baseline, 1 month, 3 months and 6 months post-surgery) and various clinical, radiographic and histological parameters were used to assess the outcomes. The results are organized into categories of soft tissue healing, hard tissue healing and overall implant success. Table 1 (see PDF) shows the GI scores for both groups at Baseline 1 month, 3 months and 6 months post-surgery. The baseline data shows that both groups had similar GI scores before surgery, with no significant differences. After surgery, the PRF group demonstrated better gingival health at all-time points, with significantly lower GI scores compared to the control group. Statistically significant differences were observed at 1 month (p = 0.002) and 3 months (p = 0.01). Table 1 (see PDF) presents the GI scores for both groups at 1 month, 3 months and 6 months post-surgery. The PRF group had lower GI scores, indicating better gingival health at all-time points, with statistically significant differences observed at 1 month (p = 0.002) and 3 months (p = 0.01). The Plaque Index (PI) scores, shown in Table 2 (see PDF), also reflected improved plaque control in the PRF group compared to the control group. Table 2 (see PDF) shows the PI scores for both groups at Baseline, 1 month, 3 months and 6 months. At baseline, the PI scores were similar in both groups. The PRF group showed a significant reduction in plaque accumulation compared to the control group, with statistically significant differences observed at 1 month (p = 0.005) and 3 months (p = 0.02). Table 3 (see PDF) shows the AL measurements for both groups at Baseline, 1 month, 3 months and 6 months. The baseline data indicates no significant difference between the two groups. The PRF group had a more stable attachment level at all-time points, with statistically significant differences observed at 1 month (p = 0.01) and 3 months (p = 0.02). The PRF group showed less attachment loss and the differences were statistically significant at 1 month (p = 0.01) and 3 months (p = 0.02). Radiographic analysis, including periapical radiographs and CBCT scans, was used to evaluate bone healing around the implant site. Table 4 (see PDF) presents the bone level measurements for both groups at Baseline, 1 month, 3 months and 6 months.

## Bone level measurements:

Table 4 (see PDF) shows the bone level measurements for both groups at Baseline, 1 month, 3 months and 6 months. At baseline, the bone levels were similar between the two groups. The PRF group exhibited superior bone level preservation at all-time points, with statistically significant differences observed at 1 month (p = 0.03), 3 months (p = 0.04) and 6 months (p = 0.02). The PRF group exhibited better bone level preservation, with statistically significant differences observed at 1 month (p = 0.03), 3 months (p = 0.04) and 6 months (p = 0.02). Bone Density was measured using Hounsfield units from the CBCT scans. Table 5 (see PDF) shows the average bone density values at 6 months post-surgery, indicating a greater increase in bone density in the PRF group. The PRF group showed a significantly higher bone density (650 HU) compared to the control group (560 HU), with a p-value of 0.02. The overall success of the implants was determined by clinical outcomes, such as implant stability, signs of infection and radiographic evidence of osseointegration. Table 6 (see PDF) presents the implant survival rates at 6 months for both groups. The PRF group had a higher implant survival rate (98%) compared to the control group (92%), with the difference being statistically significant (p = 0.04). The results of this study indicated that the use of PRF in dental implant procedures significantly improved both soft and hard tissue healing. The PRF group showed better gingival health, as evidenced by significantly lower Gingival Index (GI) and Plaque Index (PI) scores compared to the control group. Furthermore, the PRF group demonstrated more stable attachment levels around the implant site, indicating better soft tissue stability. In terms of hard tissue healing, the PRF group exhibited superior bone level preservation, with significant improvements in bone density and less bone loss at all-time points compared to the control group. The radiographic evaluations, including CBCT scans, confirmed that the PRF group had better osseointegration and bone remodeling. Overall, the implants placed with PRF demonstrated a higher success rate, with fewer complications and improved healing compared to those placed without PRF. These findings suggest that PRF could be a valuable adjunct in dental implant procedures, promoting faster healing, improving tissue responses and enhancing long-term implant success.

## Discussion:

The study provided a comparison of implants placed with and without Platelet Rich Fibrin (PRF). It showed that the PRF augmented group showed better soft tissue indicators (Gingival Index, Plaque Index, Attachment Level) and better results for the hard tissue (bone level preservation, increased bone density and higher survival rate). This supports the previous literature on PRF use in implant dentistry and also expands it. In the following text I contextualized the results within the framework of a least six previous studies and pointed out consistencies and differences, along with their relevance for clinical practice and future studies. According to Öncü *et al.* (2015) [14], the application of PRF (Platelet-rich Fibrin) around implants produced substantially greater Implant Stability Quotient (ISQ) values at 1 week and 4 weeks compared to implants without PRF, which suggests a more rapid early osseointegration. This explains our observation of improved early bone level outcomes in the PRF group and highlights the positive impact of PRF on the acceleration of implant stability. PRF was shown to improve implant stability and accelerate bone healing following implant surgeries without any additional interventions (Guan *et al.* 2023) [15]. Our hard tissue results also show, similarly to the mentioned meta-analysis, that the bone density at 6 months and 6 months postoperatively was significantly greater in the PRF group and bone level loss was less at 6 months postoperatively. Hence, it is evident that PRF continues to have a positive influence on osseointegration. In a study by Azangookhiavi *et al.* (2024) [16], a randomized clinical trial comparing alveolar ridge preservation with PRF versus freeze dried bone allograft (FDBA) found significantly less gingival recession in the PRF group at 6 and 12 months, but no significant difference in marginal bone loss between groups. In our study, soft tissue behaviour (gingival/attachment indices) shows significant improvements with PRF, whereas hard tissue differences (bone levels) also reached significance. The divergence may reflect the implant-specific context of our trial versus the extraction/ARP context of Azangookhiavi *et al.* but it confirms that PRF offers a soft-tissue advantage.

A review by Al Maawi *et al.* (2021) [17] examined the effectiveness of PRF in socket healing and found that PRF significantly improved soft tissue healing in the first 1 week post extraction and reduced bone dimensional loss at 8 15 weeks, though long term implant outcomes remained unclear. Our study similarly found early soft and hard tissue benefits with PRF and extends evidence into the 6 month period, supporting the argument for a lasting effect in the implant setting. According to the narrative review by Zwittnig *et al.* (2022) [18], PRF shows promising benefits for both soft and hard tissue regeneration in implantology, but highlighted heterogeneity in methods, outcome metrics and follow up periods in the literature. Our study contributes by using standardized indices across soft and hard tissues with a defined follow up, thereby strengthening evidence with an integrated methodology. A recent systematic review by Giammarinaro *et al.* (2025) [19] focused on soft tissue augmentation around implants and found that PRF significantly increased keratinised mucosa width and soft tissue thickness compared with controls, though free gingival grafts still outperformed it marginally. This complements our soft tissue findings: though we did not measure keratinised mucosa width specifically, improved soft tissue health (via GI, PI, AL) in the PRF group suggests improved mucosal and peri implant tissue quality. The consistent soft tissue improvement with PRF aligns well with prior literature. Moreover, our demonstration of statistically significant bone level preservation and higher bone density at 6 months adds to the relatively fewer studies reporting mid term hard tissue benefits in the implant setting. The improved survival rate (98 % vs 92 %) may suggest that these tissue level improvements translate into clinically meaningful outcomes over the early post operative period.

## Conclusion:

The use of Platelet-Rich Fibrin (PRF) in dental implant procedures significantly improves both soft and hard tissue healing, promoting better gingival health, bone preservation and implant stability. Thus, we show the clinical benefits of PRF as an adjunct in implantology. Further long-term studies are needed to confirm the sustained effects of PRF on implant success and tissue regeneration.

## References

[1] Kamalaksh VS (2012). Journal of Interdisciplinary Dentistry.

[2] Prasad P (2024). J Pharm Bioallied Sci..

[3] Pandya DJ (2025). Bulletin of Stomatology and Maxillofacial Surgery..

[4] Dutta SR (2020). Natl J Maxillofac Surg..

[5] Bahadur R (2024). J Dent Panacea.

[6] Acerra A (2025). J Clin Med..

[7] Giammarinaro E (2025). BMC Oral Health..

[8] Pavlovic V (2016). Open Med (Wars)..

[9] Costa R (2025). Biomedicines..

[10] Parithimarkalaignan S (2013). J Indian Prosthodont Soc..

[11] Stiller M (2015). Int J Implant Dent..

[12] Tabassum S (2022). Int J Health Sci (Qassim)..

[13] Damsaz M (2020). Front Surg..

[14] Oncu E (2015). Int J Oral Maxillofac Implants..

[15] Guan S (2023). Heliyon..

[16] Azangookhiavi H (2024). BMC Oral Health..

[17] Al-Maawi S (2021). Int J Implant Dent.

[18] Zwittnig K (2022). Transfus Med Hemother..

[19] Giammarinaro E (2025). BMC Oral Health.

